# X Inactivation and Progenitor Cancer Cells

**DOI:** 10.3390/cancers3022169

**Published:** 2011-04-26

**Authors:** Ruben Agrelo

**Affiliations:** Research Institute of Molecular Pathology, Dr. Bohr-Gasse 7, 1030 Vienna, Austria; E-Mail: agrelo@imp.ac.at; Tel.: +43179730; Fax: +4317987153

**Keywords:** X inactivation, *Xist* RNA, chromatin, cancer, epigenetics

## Abstract

In mammals, silencing of one of the two X chromosomes is necessary to achieve dosage compensation. The 17 kb non-coding RNA called *Xist* triggers X inactivation. Gene silencing by *Xist* can only be achieved in certain contexts such as in cells of the early embryo and in certain hematopoietic progenitors where silencing factors are present. Moreover, these epigenetic contexts are maintained in cancer progenitors in which SATB1 has been identified as a factor related to *Xist*-mediated chromosome silencing.

## 1. Introduction

The genome contains all the information required to generate the different cell types that comprise an organism. In multicellular organisms, regulation of cell identity and cell proliferation is exquisitely coordinated to produce the different cell types that form different tissues and organs.

In particular, embryonic stem (ES) cells can give rise to a broad range of specialized cells. The flexibility to regulate gene expression patterns in parallel with more dynamic chromatin behavior provides these cells with the necessary developmental plasticity.

A variety of adult stem cells that occupy different cell niches are responsible for the maintenance of tissues and organs. Furthermore, cell identity has to be maintained in these cell niches.

However, exactly how stem cells maintain their own cell identity in these niches, while still possessing the ability to generate the mature cells that compose an organism, is not clear. This may be partly explained by the concept of epigenetic context, which can be defined as the sum of all epigenetic pathways and mechanisms for a given cell or cell type. Depending on its state of differentiation, the cell has a certain epigenetic context that can either prevent or allow it to change its identity. When the cell differentiates, the epigenetic programming of differential gene expression based on different epigenetic mechanisms (e.g., DNA methylation) constantly changes.

When determining cell identity, the “marks” that can be used to define these different epigenetic contexts need to be identified.

The presence of two X chromosomes (XX) in female mammals and one (XY) in males raises the question of how to equalize the X linked genes. This is adjusted by chromosome-wide transcriptional silencing of one of the two X chromosomes in females [1]. This chromosome wide-silencing is initiated by *Xist*, which is a 17 kb untranslated RNA that coats the X chromosome in *cis* [1].

Interestingly, in mouse ES cells, the transition from an active X chromosome to an inactive X chromosome (Xi) can be followed [2]. X chromosome inactivation is divided into an *INITIATION* and a *MAINTENANCE* phase. Using genetic manipulation of *Xist* expression, *Xist* was shown to have X chromosome silencing function only in a defined epigenetic window (48–72 h) after ES cells induced differentiation [2]. Moreover, within this window, gene silencing is reversible and genes will be reactivated once *Xist* expression is lost [2].

Upon differentiation, and after the initiation phase of X inactivation, *Xist* RNA coating is not essential for the maintenance of X inactivation. In the maintenance phase, Xi becomes subject to further modifications such as DNA methylation and histone hypoacetylation, thus keeping genes repressed without needing the constant expression of *Xist* [2-4].

Thus, the shift from *Xist*-dependent initiation to stabilization of the inactive state could be regarded as a change of epigenetic context. As a consequence, it can be used as a “mark” to analyze changes in the epigenetic contexts of different cells during development [5].

Previous work has demonstrated that the silencing function of *Xist* is re-established in immature hematopoietic precursors [6]. However, whether such a transition in the *Xist* silencing ability exists in neoplastic cells is not clear. The present review discusses recent findings showing that the pathway that enables the silencing function of *Xist* is re-established in lymphoma cells [7]. Moreover, the review discusses how the study of epigenetic context transitions in cancer cells allowed the identification of the special AT-rich binding protein SATB1 as a silencing factor [7].

## 2. A Particular Epigenetic Context of Progenitors Defined by *Xist* Silencing Ability Is Maintained in Neoplastic Cells

Although X inactivation is initiated in the cells of the early embryo, experiments using tetracycline-inducible *Xist* transgenes showed the presence of cells in the adult mouse in which *Xist* can initiate gene silencing [6]. These cells are lineage-restricted progenitors within the hematopoietic system, and this phenomenon has been clearly established for preB cells and CD4+ and CD8+ double positive T-cells [6].

Interestingly, the hematopoietic stem cell (HSC) and mature blood cells do not have the cellular context for *Xist*-mediated silencing [6], which indicates that specific pathways used in the early embryo are reactivated transiently during the differentiation of the HSC. It could be argued that well-defined epigenetic pathways are shared among the early embryo and some kind of progenitor cells in which *Xist* can initiate chromosome-wide silencing [5]. A recent study demonstrated that *Xist* can trigger gene silencing in certain tumor cells [7]. Tumor formation in mice was induced by the expression of an oncogenic human lymphoma kinase fusion protein (NPM-ALK) that is expressed in the T cell compartment. This protein is generated from the fusion of the anaplastic lymphoma kinase (ALK) and the nucleophosmin gene (NPM/B23), and it is produced by a t(2;5) translocation in human anaplastic large cell lymphoma [8,9]. In this tumor model, ectopic induction of *Xist* expression caused X inactivation and suppressed tumor development [7].

**Figure 1.**
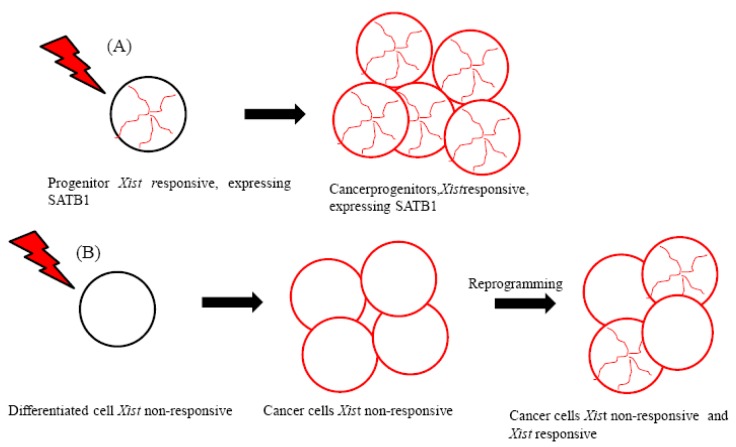
Epigenetic progenitor context A) *Xist* can silence the X chromosome in lymphoma cells arrested in a progenitor stage (e.g., double positive cells) and expressing SATB1 (in red). B) Other tumors such as breast cancer cells and cancer cells unresponsive to *Xist* may reprogram their genome, thus gaining metastatic potential. These malignant progenitors may express SATB1 and may be *Xist*-responsive.

Lymphoma cells derived from progenitors therefore maintain the epigenetic context that enables the initiation of *Xist*-mediated gene silencing (Figure 1A). These cells have been suggested to express silencing factors [7]. The special AT-rich binding protein (SATB1) was identified as an initiation factor for *Xist*-mediated silencing through a comparison of the expression profiles of *Xis*t-responsive and *Xist-*resistant tumors [7]. Because SATB1 expression was found to be restricted in development, it has been proposed to characterize the cellular context for *Xist*-mediated silencing in ES cell differentiation and in T lymphocyte development [7].

SATB1 is a DNA binding protein that facilitates the organization of chromatin structure through its interaction with AT-rich DNA sequences.

SATB1 was first described in T cells, where SATB1 forms a network that overlaps the base of chromatin loops, circumscribing heterochromatin and regulating gene expression in a coordinate manner [10]. Moreover, it has the ability to recruit chromatin remodeling complexes to these anchorage sites, and is therefore capable of regulating histone modifications or nucleosomal positioning over large regions [11]. In addition, SATB1 activity is regulated by posttranslational modifications [12-14].

Recent and interesting work has shown that some of the genes SATB1 represses are upregulated by WNT signals [15]. The binding of WNT soluble proteins to their cell surface receptors initiates a signaling cascade that ultimately results in the accumulation of β-catenin in the nucleus, where it can bind and activate different transcription factors. In the absence of WNT signaling, β-catenin is phosphorylated and degraded. Direct interaction between SATB1 and β-catenin in Th2 cells has been detected. Moreover, β-catenin causes an increase in DNA binding by SATB1 by promoting its hypoacetilation (a modification that increases SATB1 affinity for DNA). Interestingly, β-catenin also binds to the same gene. Although SATB1 binds to DNA and subsequently recruits β-catenin to the DNA, once β-catenin is bound to SATB1 it can recruit additional proteins to help stimulate gene expression. β-catenin can modify the function of SATB1 from a repressor to an activator of gene function. In future studies, it would be interesting to investigate whether WNT signaling can influence *Xist* mediated gene silencing.

In summary, *Xist*-mediated gene silencing capacity is preserved in cancer progenitors in which SATB1 has been identified as a silencing factor.

### SATB1 and Its Role in Xist-Mediated Silencing

The X chromosome comprises of a gene-rich outer rim and an internal core containing silenced non-genic sequences [5]. Upon silencing, genes are moved into the chromosome territory in a manner that is dependent on repeat A of *Xist* [16]. Immunolocalization studies in CD4^+^andCD8^+^ T cells in which SATB1 shows a ring- or cage-like staining pattern suggest that the sites where the SATB1 protein is localized do not overlap with *Xist* RNA domains. However, the induction of *Xist* RNA expression can change SATB1 localization patterns and SATB1 can influence *Xist* RNA localization. Based on these observations, it has been proposed that *Xist* can pull genes into the repressive compartment for gene silencing and that SATB1 may act as an anchor when this chromosome reorganization occurs. Interestingly, certain genes known as escapers remain external [7].

Moreover, it has been suggested that in the absence of SATB1, genes may be released to loop out from the *Xist* domain, and therefore oscillate between the *Xist* silencing domain and the transcription factories domain [17]. The association with transcription factories therefore results in gene transcription, and as a consequence, gene activity will continue despite the generation of a *Xist* RNA silencing domain [17]. The above model can explain why *Xist* is not able to silence outside narrow windows where SATB1 is expressed.

## 3. A Closer Look at This Particular Tumor Cell Context

Whether *Xist*-responsive cell types are exclusive to the embryo and the hematopoietic system, or if other adult stem cell niches such as skin are also characterized by re-establishing an appropriate epigenetic context, has not been elucidated. However, data suggest that the *Xist*-silencing pathway can be active in a wide variety of tumors. *XIST* expression is present in human testicular germ cell tumors, which is unusual considering that *XIST* is not normally expressed in male cells [18-20]. Moreover, the existence of multiple X chromosomes suggests that at a certain stage of tumor development, X inactivation must have been initiated [18-20]

On the other hand, an ectopic human *XIST* transgene has been found to induce chromosome inactivation in human HT-1080 fibrosarcoma cells [21] where SATB1 is expressed [22].

Importantly, SATB1 may be necessary for the epigenetic transition and reorganization of the genome in breast cancer cells that become highly metastatic [23-26] (Figure 1B). Supporting the role of SATB1 in promoting metastasis, SATB1 expression has been associated with the development and metastasis of bladder urothelial carcinoma and gastric carcinoma [27,28].

However, the findings of a recent study were in disagreement with previous results showing that SATB1 promotes metastasis in breast cancer [24]. Recently, it was suggested that this discrepancy may be due to differences in experimental conditions between these studies [25].

Taken together, these observations suggest that the context for *Xis*t-mediated silencing might be found in a wider range of progenitors in different tissues, and in different cancer cells that may originate from them.

Following this line of thought, similarities between gene expression patterns of ES cells and poorly differentiated aggressive forms of cancer have been observed by bioinformatic analysis of expression data for a set of tumors [29]. Furthermore, chromatin organization and composition similarities between ES cells and tumor cells have also been reported [30].

As a result, it could be argued that tumor cells use chromatin regulation and epigenetic pathways that are normally active in ES cells.

## 4. Concluding Remarks

Analysis of the *Xist* gene silencing pathway could improve our understanding of the pathways that are active in narrow developmental windows in which cell identity is unstable and are conserved in neoplastic cells. One reason for restricting the activity of the silencing pathway could be to safeguard against illegitimate changes of cell identity that could lead to malignant transformation. The neoplastic cell could exploit these pathways, for example, to gain metastasizing potential. However, it still remains a challenge to analyze the *Xist*-silencing function in a wider spectrum of tumors to gain knowledge on how general the silencing context is in tumor formation. Furthermore, the possibility of classifying tumors according to *Xist* responsiveness is questionable. If possible, it would be important to characterize these tumors for metastatic potential and resistance to chemotherapy. Following this line of thought, recent observations have revealed that SATB1 expression is upregulated in multidrug-resistant breast cancer cells [31].

Further studies of these pathways could provide important information for the development of new therapies.

## References

[1] Payer B., Lee J.T. (2008). X chromosome dosage compensation: How mammals keep the balance. Ann. Rev. Genet..

[2] Wutz A., Jaenisch R. (2000). A shift from reversible to irreversible X inactivation is triggered during ES cell differentiation. Mol. Cell.

[3] Brown C.J., Willard H.F. (1994). The human X-inactivation centre is not required for maintenance of X-chromosome inactivation. Nature.

[4] Csankovszki G., Panning B., Bates B., Pehrson J.R., Jaenisch R. (1999). Conditional deletion of Xist disrupts histone macroH2A localization but not maintenance of X inactivation. Nat. Genet..

[5] Wutz A. (2007). Xist function: Bridging chromatin and stem cells. Trends Genet..

[6] Savarese F., Flahndorfer K., Jaenisch R., Busslinger M., Wutz A. (2006). Haematopoietic precursor cells transiently reestablish permissiveness for X inactivation. Mol. Cell. Biol..

[7] Agrelo R., Souabni A., Novatchkova M., Haslinger C., Leeb M., Komnenovic V., Kishimoto H., Gresh L., Kohwi-Shigematsu T., Kenner L. (2009). SATB1 defines the developmental context for gene silencing by Xist in lymphoma and embryonic cells. Dev. Cell.

[8] Chiarle R., Voena C., Ambrogio C., Piva R., Inghirami G. (2008). The anaplastic lymphoma kinase in the pathogenesis of cancer. Nat. Rev. Cancer.

[9] Morris S.W., Kirstein M.N., Valentine M.B., Dittmer K.G., Shapiro D.N., Saltman D.L., Look A.T. (1994). Fusion of a kinase gene, ALK, to a nucleolar protein gene, NPM, in non-Hodgkin's lymphoma. Science.

[10] Cai S., Lee C.C., Kohwi-Shigematsu T. (2006). SATB1 packages densely looped, transcriptionally active chromatin for coordinated expression of cytokine genes. Nat. Genet..

[11] Yasui D., Miyano M., Cai S., Varga-Weisz P., Kohwi-Shigematsu T. (2002). SATB1 targets chromatin remodelling to regulate genes over long distances. Nature.

[12] Pavan Kumar P., Purbey P.K., Sinha C.K., Notani D., Limaye A., Jayani R.S., Galande S. (2006). Phosphorylation of SATB1, a global gene regulator, acts as a molecular switch regulating its transcriptional activity *in vivo*. Mol. Cell..

[13] Purbey P.K., Singh S., Notani D., Kumar P.P., Limaye A.S., Galande S. (2009). Acetylation-dependent interaction of SATB1 and CtBP1 mediates transcriptional repression by SATB1. Mol. Cell. Biol..

[14] Tan J.A., Sun Y., Song J., Chen Y., Krontiris T.G., Durrin L.K. (2008). SUMO conjugation to the matrix attachment region-binding protein, special AT-rich sequencebinding protein-1 (SATB1), targets SATB1 to promyelocytic nuclear bodies where it undergoes caspase cleavage. J. Biol. Chem..

[15] Notani D., Gottimukkala K.P., Jayani R.S., Limaye A.S., Damle M.V., Mehta S., Purbey P.K., Joseph J., Galande S. (2010). Global regulator SATB1 recruits beta-catenin and regulates T(H)2 differentiation in Wnt-dependent manner. PLoS Biol..

[16] Wutz A., Rasmussen T., Jaenisch R. (2002). Chromosomal silencing and localization are mediated by different domains of Xist RNA. Nat. Genet..

[17] Brockdorff N. (2009). SAT in silence. Dev. Cell.

[18] Kawakami T., Okamoto K., Ogawa O., Okada Y. (2004). XIST unmethylated DNA fragments in male-derived plasma as a tumour marker for testicular cancer. Lancet.

[19] Kawakami T., Okamoto K., Sugihara H., Hattori T., Reeve A.E., Ogawa O., Okada Y. (2003). The roles of supernumerical X chromosomes and *XIST* expression in testicular germ cell tumors. J. Urol..

[20] Looijenga L.H., Gillis A.J., van Gurp R.J., Verkerk A.J., Oosterhuis J.W. (1997). X inactivation in human testicular tumors. *XIST* expression and androgen receptor methylation status. Am. J. Pathol..

[21] Hall L.L., Byron M., Sakai K., Carrel L., Willard H.F., Lawrence J.B. (2002). An ectopic human *XIST* gene can induce chromosome inactivation in postdifferentiation human HT-1080 cells. Proc. Natl. Acad. Sci. USA.

[b22-cancers-03-02169] 22.Agrelo, R. Institut Pasteur de Montevideo, Mataojo 2020, 1140 Montevideo, Uruguay, Unpublished results, 2011.

[23] Han H.J., Russo J., Kohwi Y., Kohwi-Shigematsu T. (2008). SATB1 reprogrammes gene expression to promote breast tumour growth and metastasis. Nature.

[24] Hnatyszyn H.J., Seo P., Clarke J., Ward T., Lippman M. (2010). The role of SATB1 in breast cancer pathogenesis. J. Natl. Cancer Inst..

[25] Kohwi-Shigematsu T., Han H.J., Russo J., Kohwi Y. (2010). Re: The role of SATB1 in breast cancer pathogenesis. J. Natl. Cancer Inst..

[26] Richon V.M. (2008). A new path to the cancer epigenome. Nat. Biotechnol..

[27] Lu X., Cheng C., Zhu S., Yang Y., Zheng L., Wang G., Shu X., Wu K., Liu K., Tong Q. (2010). SATB1 is an independent prognostic marker for gastric cancer in a Chinese population. Oncol. Rep..

[28] Liu C.X., Wen Y., Xu K., Zheng S.B., Xu Y.W., Chen B.S. (2010). Expression of special AT-rich sequence-binding protein in bladder urothelial carcinoma and its clinical significance. Nan Fang Yi Ke Da Xue Xue Bao.

[29] Ben-Porath I., Thomson M.W., Carey V.J., Ge R., Bell G.W., Regev A., Weinberg R.A. (2008). An embryonic stem cell-like gene expression signature in poorly differentiated aggressive human tumors. Nat. Genet..

[30] Ohm J.E., McGarvey K.M., Yu X., Cheng L., Schuebel K.E., Cope L., Mohammad H.P., Chen W., Daniel V.C., Yu W. (2007). A stem cell-like chromatin pattern may predispose tumor suppressor genes to DNA hypermethylation and heritable silencing. Nat. Genet..

[31] Li Q.Q., Chen Z.Q., Xu J.D., Cao X.X., Chen Q., Liu X.P., Xu Z.D. (2010). Overexpression and involvement of special AT-rich sequence binding protein 1 in multidrug resistance in human breast carcinoma cells. Cancer Sci..

